# Nitrogen-dependent coordination of cell cycle, quiescence and TAG accumulation in Chlamydomonas

**DOI:** 10.1186/s13068-019-1635-0

**Published:** 2019-12-23

**Authors:** Tomomi Takeuchi, Christoph Benning

**Affiliations:** 1 Department of Biochemistry and Molecular Biology, Michigan State University, East Lansing, MI 48824 USA; 2 Department of Energy-Plant Research Laboratory, Michigan State University, East Lansing, MI 48824 USA; 3 Department of Plant Biology, Michigan State University, East Lansing, MI 48824 USA

**Keywords:** Chlamydomonas, Quiescence, Nitrogen deprivation, Triacylglycerols, Cell cycle, DREAM complex, TOR, SnRK/CKIN, Biofuel, Biomass

## Abstract

Microalgae hold great promises as sustainable cellular factories for the production of alternative fuels, feeds, and biopharmaceuticals for human health. While the biorefinery approach for fuels along with the coproduction of high-value compounds with industrial, therapeutic, or nutraceutical applications have the potential to make algal biofuels more economically viable, a number of challenges continue to hamper algal production systems at all levels. One such hurdle includes the metabolic trade-off often observed between the increased yields of desired products, such as triacylglycerols (TAG), and the growth of an organism. Initial genetic engineering strategies to improve lipid productivity in microalgae, which focused on overproducing the enzymes involved in fatty acid and TAG biosynthesis or inactivating competing carbon (C) metabolism, have seen some successes albeit at the cost of often greatly reduced biomass. Emergent approaches that aim at modifying the dynamics of entire metabolic pathways by engineering of pertinent transcription factors or signaling networks appear to have successfully achieved a balance between growth and neutral lipid accumulation. However, the biological knowledge of key signaling networks and molecular components linking these two processes is still incomplete in photosynthetic eukaryotes, making it difficult to optimize metabolic engineering strategies for microalgae. Here, we focus on nitrogen (N) starvation of the model green microalga, *Chlamydomonas reinhardtii*, to present the current understanding of the nutrient-dependent switch between proliferation and quiescence, and the drastic reprogramming of metabolism that results in the storage of C compounds following N starvation. We discuss the potential components mediating the transcriptional repression of cell cycle genes and the establishment of quiescence in Chlamydomonas, and highlight the importance of signaling pathways such as those governed by the target of rapamycin (TOR) and sucrose nonfermenting-related (SnRK) kinases in the coordination of metabolic status with cellular growth. A better understanding of how the cell division cycle is regulated in response to nutrient scarcity and of the signaling pathways linking cellular growth to energy and lipid homeostasis, is essential to improve the prospects of biofuels and biomass production in microalgae.

## Background

The use of algae as a potential source of renewable fuel, animal feeds in addition to nutrients and pharmaceuticals for human health has been recognized and exploited for decades. Both micro- and macro-algae constitute a diverse group of primarily aquatic photosynthetic organisms with varying levels of complexity, and their natural biochemical compositions (e.g., high contents of oil, carbohydrates, proteins, sugars, vitamins, pigments, or minerals) make them uniquely suitable for different commercial purposes. In addition to their relevance in agriculture as fertilizers, soil conditioners and livestock feeds, algae provide many nutrients essential for human health, including vitamins, minerals, anti-oxidants, and polyunsaturated fatty acids such as docosahexaenoic acids and eicosapentaenoic acids [1–3]. Furthermore, algae-derived products are also used as gelling agents and stabilizers in various food products, cosmetics and pharmaceuticals [1–3]. Over thirty recombinant proteins have been successfully produced in microalgae to date, including hormones, enzymes, antibodies, vaccines and immunotoxins, highlighting the biotechnical utilities and potentials of these organisms [4–7]. In the past few decades, microalgae have garnered renewed interests as alternative feedstocks for the sustainable production of biofuels, in the forms of biodiesel, bioethanol, biogas and hydrogen. Many microalgae are able to grow rapidly to high cell densities using CO_2_ or other provided carbon (C) sources, can be cultivated using nonarable land and water sources not suited for agriculture, and accumulate more triacylglycerols (TAG) per dry weight or per unit area than agricultural oil crops [8–12]. Because algae maintain high productivity in nutrient-rich waters, they can be used to remove excess nutrients from waste water and mitigate fertilizer runoff from farms, while simultaneously yielding biomass and precursors for the production of biofuels [13–15]. In addition, the use of industrial flue gas as a source of C, the biorefinery-based approach to biofuel production, and the concurrent cultivation of high-value compounds were also proposed as a potential means to further lower the cost of algal biofuels [8–10, 12, 16].

Although the production of sustainable energy and economically valuable compounds from algae hold great promises, a number of hurdles persist. These include the species-dependent recalcitrance to various genetic manipulations, suboptimal utilization and conversion of light energy and CO_2_ to biomass due to light saturation and or photoinhibition, limited light penetrance in the culture, undesirable contamination, and high costs ultimately associated with sustaining optimal growth and metabolic outputs, as well as high costs of extraction and processing [8, 11, 12, 17, 18]. Another major impediment that prevents algal biofuels from becoming a competitive alternative to fossil petroleum on a commercial scale involves the inverse relationship between the yield of cellular products and the growth of the organism. A plethora of abiotic stresses such as nutrient deprivation, extreme light conditions and changes in temperature, salinity or pH is known to induce the accumulation of sought-after molecules in algae, including TAG, hydrogen, and carotenoids like β-carotene and astaxanthin [9, 18–25]. However, the increase in these compounds often comes at the expense of inhibited growth, resulting in the considerable reduction of biomass. A two-stage cultivation strategy, where the algal cells are subjected to stress only after a period of optimal growth and accumulation of biomass, has been proposed and tested to circumvent this problem [26–28], but this production method is still costly due to its extended requirement for time and the inherent complexity in monitoring and optimizing the production process.

Genetic engineering strategies in algae, which aimed to alter the expression levels of genes encoding individual enzymes involved in lipid metabolism, TAG biosynthesis and catabolism, or other competing C metabolic pathways, have seen mixed outcomes in achieving the optimal balance between lipid productivity and growth [29, 30]. Recent approaches targeting transcription factors or signaling pathways that regulate C and growth metabolisms in algae appear to achieve more consistent successes in increasing TAG content with little or no compromise in cellular growth and proliferation by simultaneously modifying multiple components of a metabolic pathway [31–36]. However, the regulatory components and signaling networks coordinating the allocations of C towards storage and growth are still not well characterized in photosynthetic eukaryotes, which continues to hamper the metabolic engineering efforts for algae. Therefore, a better understanding of the molecular mechanisms by which metabolism and growth are regulated and coupled is necessary. Here, the nutrient-dependent transitions between the cell division and quiescence cycles, the shifts in metabolism towards the synthesis of C storage compounds following nutrient starvation, and the potential molecular components mediating the cessation of growth and entry into quiescence under stress conditions are discussed focusing on nitrogen (N) starvation in the model green microalgae, *Chlamydomonas reinhardtii*, as a reference. Our current understanding of the signaling pathways integrating the changes in metabolism and the cell division cycle in response to nutrient availability in algae is presented, followed by concluding remarks on the potential biotechnological implications of the presented concepts.

## Chlamydomonas as a model to study key life-cycle transitions

**Fig. 1:**
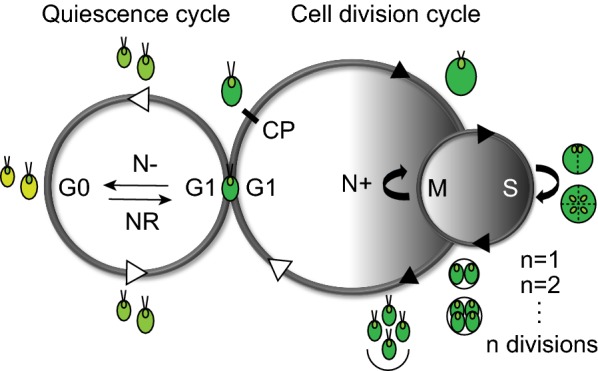
The intersection between the cell division and the quiescence cycles in Chlamydomonas. The circles to the right represent the cell division cycle of Chlamydomonas characterized by multiple fissions, where the cells increase in volume during a prolonged growth (G1) phase during the light phase (white left half), followed by rapid rounds of S/M (DNA synthesis and mitosis) cycles during the dark phase (shaded right half) to give rise to 2^*n*^ daughter cells of equal size. The commitment point (CP) represents the size-dependent checkpoint. Upon the passage of CP, the cells commit to completing at least one round of division even when the light or nutrients are subsequently withdrawn. The left circle represents the quiescence cycle, where the cells cease further growth and division with 1C (one copy) chromatin content. The entry into and exit from the quiescence (G0) are controlled by the availability of nutrients, such as nitrogen (N), and the respective changes in chlorophyll content of Chlamydomonas cells are depicted by different shades of green. The coupling of these two opposing cycles occurs during the post-mitotic resting stage or G1 phase prior to the passage of CP. Cell cycle-dependent steps are represented by the black arrow heads, while the nutrient-dependent steps are represented by the white arrow heads. N+: N-replete growth; N−: N deprivation; NR: N refeeding

At the cell biological level, many abiotic stresses will induce cells to accumulate storage compounds and exit the normal cell division cycle to enter an alternative reversible state known as quiescence, or G0 [37]. When the conditions are again conducive to growth, cells degrade the accumulated storage compounds, exit quiescence and reenter the cell division cycle [38]. Chlamydomonas serves as a particularly excellent model system to study the coordination between metabolism, cell division cycle and quiescence in photosynthetic organisms for several reasons. Chlamydomonas can be grown rapidly under heterotrophic, photoautotrophic or mixotrophic conditions, depending on the research needs [39]. For instance, the growth and division of Chlamydomonas cells can be synchronized with alternating light and dark cycles when they are grown photoautotrophically, enabling the facile isolation of cells at different stages of the cell cycle [40] (Fig. 1). In addition, the life-cycle transitions between cell division to quiescence and vice versa can be discretely controlled and analyzed by selective removal or resupply of nutrient(s) in the medium (Fig. 1). Furthermore, a great number of -omics-based studies has been conducted using Chlamydomonas under different stress conditions in the past decade, and a wealth of literature on how Chlamydomonas cells reprogram their metabolism in response to nutrient shortage, such as N starvation at the levels of transcriptome, proteome and metabolome is available [41–49]. While Chlamydomonas is not typically considered a candidate alga for the production of biofuel feedstocks, Chlamydomonas cells still accumulate a significant amount of TAG just as other oleaginous algae do in response to nutrient starvation [20]. Combined with the availability of well-established molecular genetics and genomic tools (e.g., the annotated genome, transformation protocols, reverse genetic engineering tools, and mutant libraries [50–53]) and the haploid genome of Chlamydomonas during vegetative growth [39], there is a solid infrastructure for the further exploration of the regulatory link between metabolism and life-cycle transitions in this alga.

## The intersection between the cell division cycle and the quiescence cycle in Chlamydomonas

In the presence of sufficient nutrients, Chlamydomonas and many other green algae grow and divide by a modified cell cycle involving multiple fissions, where the cells go through a prolonged growth or G1 phase followed by a rapid succession of S/M (DNA synthesis and mitosis) cycles [54–56] (Fig. 1). The gap between the S and M phases (known as the G2 phase) is not observed in the cell cycle of Chlamydomonas [57]. In addition, the cell cycle of photoautotrophically grown Chlamydomonas cells synchronizes under diel conditions such that cellular growth, flagella-dependent phototaxis and light-dependent reactions of photosynthesis are maximized during the day. Processes such as the replication of DNA and cell division (i.e., S/M phase), which may benefit from the absence of potentially damaging photons and require the resorption of flagella for the basal body-mediated coordination of mitosis and cytokinesis, are timed to occur during the night [40, 55, 56, 58]. Early in G1, the newly hatched Chlamydomonas daughter cells are in a stage called pre-commitment, where the cells have not yet reached the critical size necessary to achieve competency for division. When these pre-commitment cells reach a critical volume, they pass a size-regulated checkpoint termed “Commitment”, which is a point of no return similar to “Start” in yeast and “Restriction Point” in mammalian cell cycles [40, 59–61]. Since growing Chlamydomonas cells may reach more than ten times their initial volume before the start of the S/M phase after a prolonged G1 phase, multiple rounds (*n*) of S/M cycles are necessary to produce 2^*n*^ daughters of equal size [61]. Thus, the number of S/M cycles that each mother cell undergoes is determined by its cell size such that daughters of uniform size distributions are always achieved [59, 60] (Fig. 1).

On the other hand, when faced with nutrient limitation, single-celled organisms exit from the active proliferative cycle and forego the anabolic, energy-consuming metabolism that is required for growth and division in favor of energy-saving metabolism that defines quiescence [62]. This is also the case for microalgae such as Chlamydomonas (Fig. 1). The ability of ancestral eukaryotic cells to enter a state of quiescence, maintain viability, and subsequently resume growth when the conditions improve, was likely critical, not only for their immediate survival but the subsequent evolution of species. The molecular mechanisms by which cells transition from active cell division to quiescence cycles and vice versa in response to nutrient availability are best characterized in yeast [38]. However, the capacity to orchestrate these life-cycle changes is evolutionarily conserved in many organisms. For instance, the entry into quiescence in cultured mammalian cells can be induced by serum and amino acid starvation, high cell density, and anchorage deprivation [63–67], although their proliferation is typically controlled by the developmental and contextual cues within the organism. Thus, some features of quiescent cells appear to be more universal. Although exceptions exist, these include the arrest of growth and cell division before the genome replicates, chromosome compaction, induction of autophagy, reduced rates of transcription and translation, and increased content of C storage molecules [37, 38, 62, 68].

An increasing body of work in opisthokonts as well as in Chlamydomonas suggests that quiescence is a poised and actively maintained state, where the entry into and exit from such a state represent distinct processes governed by unique signaling and gene-regulatory networks, rather than a phase of the cell division cycle or a passive inactive state [38, 67–72] (Fig. 1). In the opisthokont models of quiescence, the intersection between the cell division cycle and the so called quiescence cycle is thought to occur early in the G1 phase or during the “restrictive window” following the completion of a previous cell cycle before the cells pass their respective G1 checkpoints [38, 68]. In Chlamydomonas, it is also during the G1 or the post-mitotic resting phase prior to the passage of the commitment point that the cells are faced with a decision whether to proceed with the subsequent steps of the cell division cycle or to enter an alternative quiescence cycle (Fig. 1). After passing the commitment point, Chlamydomonas cells will undergo at least one round of division even after nutrients or light are withdrawn [40, 60], likely because the completion of the cell division cycle is under the control of the intrinsic oscillation of cell cycle regulators such as cyclin-dependent kinases (CDKs) [56]. Thus, it is only when the cyclical transcriptional waves during the cell cycle cease and the cells arrive at the pre-commitment stage that they are able to enter into the quiescence cycle.

The entry into the quiescence cycle in the early G1 phase before genome replication is likely important for the maintenance of viability during quiescence and the successful reentry into the cell division cycle in response to growth-promoting cues. Because quiescent cells cannot effectively dilute out molecules such as DNA damaged by reactive oxygen species (ROS) through growth and cell division, replace them through active synthesis, or repair them by energy-costly mechanisms, the condensation of chromosomes facilitates the preservation of genomic integrity and promotes survival [37, 38, 62]. Although the transcripts and protein products of most cell cycle genes are not essential for the maintenance of quiescence and survival in mammalian quiescent cells, the repression of genes that promote cell cycle progression, including genes encoding mitotic CDKs and their associated cyclins, is critical for the appropriate exit from the cell division cycle, the establishment of quiescence and the subsequent resumption of growth [62, 73, 74]. In response to quiescence-inducing cues, the inhibitors of G1 CDKs become upregulated in various quiescent mammalian cell-lineages [62]. For instance, these inhibitors act to maintain the hematopoietic stem cells in quiescence and prevent them from inappropriately or precociously entering the cell cycle [62, 75, 76]. These functions of CDK inhibitors appear conserved also in yeast [77]. Yeast mutant cells that have lost the ability to repress certain growth and cell cycle-related genes following glucose exhaustion also have shortened lifespan and fail to successfully exit quiescence upon glucose refeeding [78]. In Chlamydomonas, the cell density of mixotrophically grown cells will approximately double using the finite reservoir of intracellular N within the first 24 h of N starvation [43, 51]. Following this increase in cell number, the expression of genes involved in cell cycle progression, DNA synthesis, and replication is substantially reduced [42], and by day 2 of N deprivation, greater than 70% of the cellular population arrests growth with 1C (one copy) chromatin content [72]. Therefore, in the face of starvation, the arrest of further growth and division prior to DNA replication during the pre-commitment phase is also likely an important factor enabling successful life-cycle transitions of Chlamydomonas.

## Cellular changes that accompany N starvation and the entry into quiescence in Chlamydomonas

**Fig. 2:**
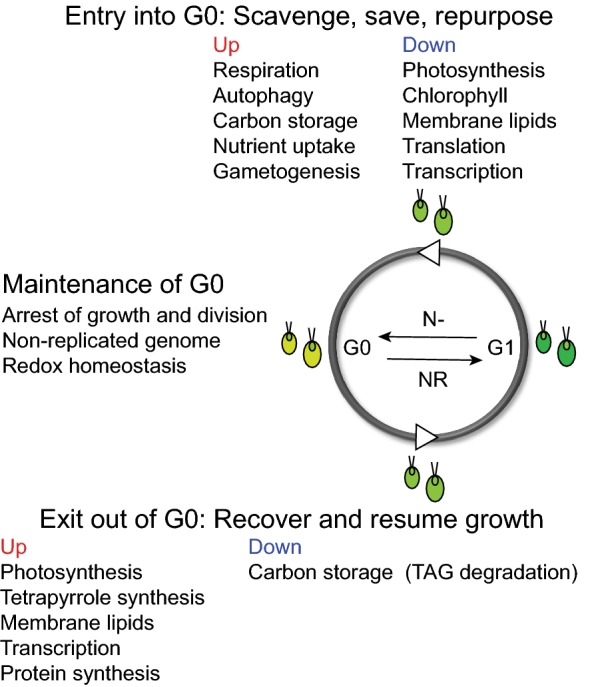
Cellular changes accompanying the entry into and exit out of quiescence in Chlamydomonas. The quiescence cycle of Chlamydomonas cells is depicted, where the cells are colored in different shades of green according to the respective changes in chlorophyll content. The summary of characteristics that Chlamydomonas cells must acquire during the entry into (following N deprivation, N−) and exit of quiescence (G0) (following N refeeding, NR) are shown. The maintenance of a quiescent state is an active process. The repression of genes associated with cell cycle progression, DNA synthesis and replication must be maintained in order to prevent the premature entry into the cell division cycle in the absence of nutrient(s), such as N. The effective management of damaging reactive oxygen species (ROS) and the achievement of redox homeostasis are necessary to promote cellular survival during the non-dividing, energy-limited state. When N becomes available, the cells that remain viable and metabolically active are able to remobilize the accumulated carbon storage, such as triacylglycerols (TAG), remodel photosynthetic membranes, and resume the synthesis of macromolecules in order to reenter the growth (G1) phase. The white arrow heads depict the nutrient-dependent nature of these steps

As the universal features of quiescence are further refined, it is becoming evident that the transition from the cell division cycle to the quiescence cycle and its reversal require the genome-wide adjustment of regulatory networks, metabolism, and intracellular structures (discussed in detail below and summarized in Fig. 2), and where applicable, necessitate the repression of alternative non-dividing cell fates such as apoptosis, senescence and differentiation [67, 68, 79]. Despite the conservation of many quiescence-associated components and processes, the generation of chemical energy from light and CO_2_ imposes another layer of complexity on the maintenance of a non-replicating, viable, and reversible state in photosynthetic organisms. While many abiotic stresses trigger algal cells to enter a quiescent state and to form lipid droplets rich in TAG, the consequences of nutrient deprivation, especially that of N, is the best studied process [26, 71]. It has been long known that N starvation induces the transcriptional program necessary for gametogenesis, during which the cells of opposite mating types differentiate into gametes capable of mating [80]. The fusion of these gametes allows for the formation of diploid zygospores, which are markedly more resilient to environmental insults than Chlamydomonas cells during vegetative growth [81, 82]. In more recent years, multiple -omics-based approaches have been successfully applied to study the systems-level responses of this alga to N deprivation, revealing the wholesale cellular reprogramming of transcriptome, proteome and metabolome that results in the accumulation of C storage and a reversible quiescent state [41–49, 71, 72, 83] (Fig. 2).

Although some responses of the starved cells are nutrient-specific, the underlying fundamental principles governing microbes under starvation can be summarized in three words—scavenge, conserve, and recycle [84]. In general, nutrient-deprived Chlamydomonas cells actively increase the scavenging and uptake of the limiting nutrient(s), curtail anabolic energy-consuming metabolism associated with growth and proliferation, and strategically repurpose nonessential macromolecules to maximize survival (Fig. 2). Although ammonium is the preferred source of N, Chlamydomonas cells can also assimilate other inorganic N-containing compounds [85]. Thus, following N deprivation, the abundance of transcripts and proteins involved in the transport and the metabolism of alternative, less favorable N sources increases almost immediately (within 0.5–1 h) [43, 85]. The cellular-wide reprogramming of metabolism occurs at the levels of transcripts and proteins to conserve energy and minimize N consumption. The levels of both cytoplasmic and chloroplast ribosomes decrease substantially [86, 87], and the total cellular contents of RNA [88] and protein [43] per cell become reduced by approximately 60% and 50%, respectively. It has been reported that the proteins whose abundance increases upon transfer of cells to N-free medium, such as those needed for N acquisition and metabolism, contain less N on average than those that decrease in abundance, highlighting the evolutionarily selected N sparing strategy to reduce the cellular demand for N when it is not readily available in the environment [43]. Chlamydomonas cells also utilize a similar conservation mechanism during sulfur (S) shortage, such that the abundant proteins under S-deficient conditions contain less S in their amino acid side chains [89]. While the genes encoding the key enzymes of glyoxylate cycle and gluconeogenesis are downregulated [42], those involved in the biosynthesis and branching of starch peak shortly after the transfer of cells to N-free medium, followed by a steady decrease in their transcript levels until the new basal level is achieved [46]. This is in contrast to genes encoding enzymes of TAG biosynthesis, whose transcript abundance gradually increases over the 2 day time course [46, 90], consistent with the observations that starch accumulation precedes the increase in TAG [45, 91, 92]. To recycle and repurpose intracellular reserves of N, the nonessential or damaged macromolecules are engulfed within a specialized double-membrane vesicle called autophagosome and are trafficked for degradation into the vacuole [93]. The induction of autophagy is one of the hallmarks of quiescent cells. Although many growth-inhibiting stresses lead to the activation of autophagy in eukaryotes [38, 93, 94], this catabolic process is also necessary for the survival and maintenance of quiescent lymphocytes and hematopoietic stem cells in mammals [62]. Yeast mutants defective in autophagy accumulate higher levels of ROS and rapidly lose viability during nutrient starvation due to their inability to remobilize amino acids and synthesize proteins necessary for stress adaptation [95–97]. A similar loss of viability was recently shown for autophagy-defective mutants of Chlamydomonas in response to deprivation of N, P (phosphorus) and S [98], suggesting the importance of this catabolic pathway for stress acclimation and cellular homeostasis of this alga [94].

Following N deprivation, Chlamydomonas cells increasingly rely on respiratory metabolism to produce energy instead of photosynthesis [43]. The cessation of chlorophyll synthesis, which is regulated both transcriptionally and post-translationally, leads to a drastic decrease in cellular chlorophyll content [43, 46, 99]. A marked multi-level downregulation of photosynthesis takes place. The abundances of many transcripts and proteins encoding the subunits of light-harvesting complexes, the cytochrome *b*_6_*f* complex, photosystems I and II, and the plastid ATP synthase complex decrease with different kinetics, ultimately leading to reduced photosynthetic capacity, efficiency and flux [42, 43, 88, 99–102]. After 2 days of N deprivation, the cellular levels of plastid membrane lipids, especially of monogalactosyldiacylglycerol (MGDG), are reduced while the TAG content increases, likely to sequester acyl groups inertly in lipid droplets as the extent of the photosynthetic membrane decreases [91, 99, 103, 104]. Most transcripts and enzymes of the Calvin–Benson cycle, especially rubisco, are reduced in abundance following 2 days of N deprivation, resulting in the increased levels of its intermediates [42, 43]. In agreement with these observations, the rates of carbon assimilation and consumption decrease significantly during N starvation [105]. Although the mRNA levels of mitochondrial respiratory complexes remain relatively stable during the 2 days of N starvation, their protein levels, along with the corresponding mitochondrial ATP synthase and cytochrome *bc1* complex components, become more abundant [43]. Consistently, the oxygen consumption increases on a protein basis, further corroborating the bioenergetic preference for respiration over photosynthesis during N deprivation [43].

The recent interest in regulatory and metabolic pathways governing TAG accumulation in microalgae has led to the identification of key enzymes responsible for the biosynthesis of TAG in Chlamydomonas [20]. Although the expression changes of many fatty acid and lipid metabolism genes are modest, notable changes in the transcript levels of genes involved in TAG biosynthesis and a number of lipases are observed following N deprivation [42, 43, 90]. The Chlamydomonas genome encodes one type I (*DGAT1*) and five type II (*DGTT1*–5) diacylglycerol acyltransferases, which catalyze the transfer of an acyl-moiety from acyl-CoA to the *sn*-3 position of diacylglycerols (DAG), and one phospholipid: DAG acyltransferase (PDAT), which catalyzes the transfer of an acyl chain at the *sn*-2 position of membrane lipids to the *sn*-3 position of DAG, resulting in the synthesis of TAG [20, 106, 107]. Among them, the transcript levels of *DGAT1*, *DGTT1*, and *PDAT* show the most significant upregulation following N deprivation [42, 43, 90]. The Chlamydomonas PDAT catalyzes the biosynthesis of TAG through its acyltransferase and acylhydrolase activities toward a broad range of acyl-lipid substrates, including galactolipids, phospholipids, cholesteryl esters and TAG [104]. The genes encoding proteins with potential roles in TAG lipolysis, such as acylglycerol lipase, *LIP1* (*Lipase 1*) with a likely role in DAG turnover [108] and those encoding the putative peroxisomal β-oxidation enzymes are concurrently downregulated [42]. Chlamydomonas cells can directly funnel exogenous acetate towards the synthesis of fatty acids and TAG following N deprivation, and the presence of acetate increases the TAG yield [42, 92, 103, 109]. Under mixotrophic conditions, over 80% of starch molecules are produced from the assimilated photosynthates or CO_2_. However, under these conditions, approximately 75% of the C used for the de novo-synthesis of fatty acids and 70% of the subsequently assembled TAG or other lipid species are derived from acetate following N starvation [105], supporting the previous 30% estimate of the contribution of membrane lipid turnover to TAG synthesis [103]. Additional fatty acids are derived from the remodeling of plastid membranes by enzymes such as PGD1 (Plastid Galactoglycerolipid Degradation 1), a lipase responsible for cleaving the acyl chains from MGDG for the synthesis of TAG [110].

Many studies have historically observed how N deprivation results in the diversion of C towards storage compounds, namely TAG and starch, at the expense of decreased growth in a number of microalgae [9, 80, 111–113]. The tight coupling and inverse relationship between TAG accumulation and proliferation have also been demonstrated in yeast, where the inhibition of cell cycle progression leads to the increased formation of lipid droplets, regardless of whether the delay is caused by drugs or mutations in genes encoding cell cycle regulators [114]. It was also recently reported that the mRNAs encoding early fatty acid synthesis enzymes (e.g., acetyl-CoA carboxylase 1, ACC1 and fatty acid synthase 1 and 2, FAS1 and 2) are translated in a cell cycle and nutrient dependent manner in yeast [115]. However, the analogous proteins in Chlamydomonas are less abundant following N deprivation than in yeast [43]. Nevertheless, different hypotheses and theories were put forward to answer the question of why algae accumulate TAG in response to growth-inhibiting stresses following entry into quiescence. The potential physiological roles of lipid droplets and TAG during stress include fatty acid storage for survival and future membrane biosynthesis, a transient reservoir of acyl groups for the remodeling of the lipids in the photosynthetic membrane, a reservoir of carotenoids for photoprotection, and a sink for excess and unused photosynthetic energy and reductants to prevent photo-oxidative damage [9, 24, 105, 110, 116–121]. The studies in yeast suggest that the availability of acetyl-CoA, a central carbon metabolite that is derived from the breakdown of C storage, is a crucial factor for the cellular exit from quiescence and reentry into the cell division cycle. These studies suggest that the rapid increase in acetyl-CoA that results upon a suitable metabolic stimulation is necessary for driving the acetylation of histones at growth regulatory genes, their activation, and consequently enable cells to exit from quiescence [122, 123]. Whether these regulatory principles apply to algae remain to be explored.

## The DREAM complex: a master transcriptional regulator of cell division cycle also in algae?

**Fig. 3:**
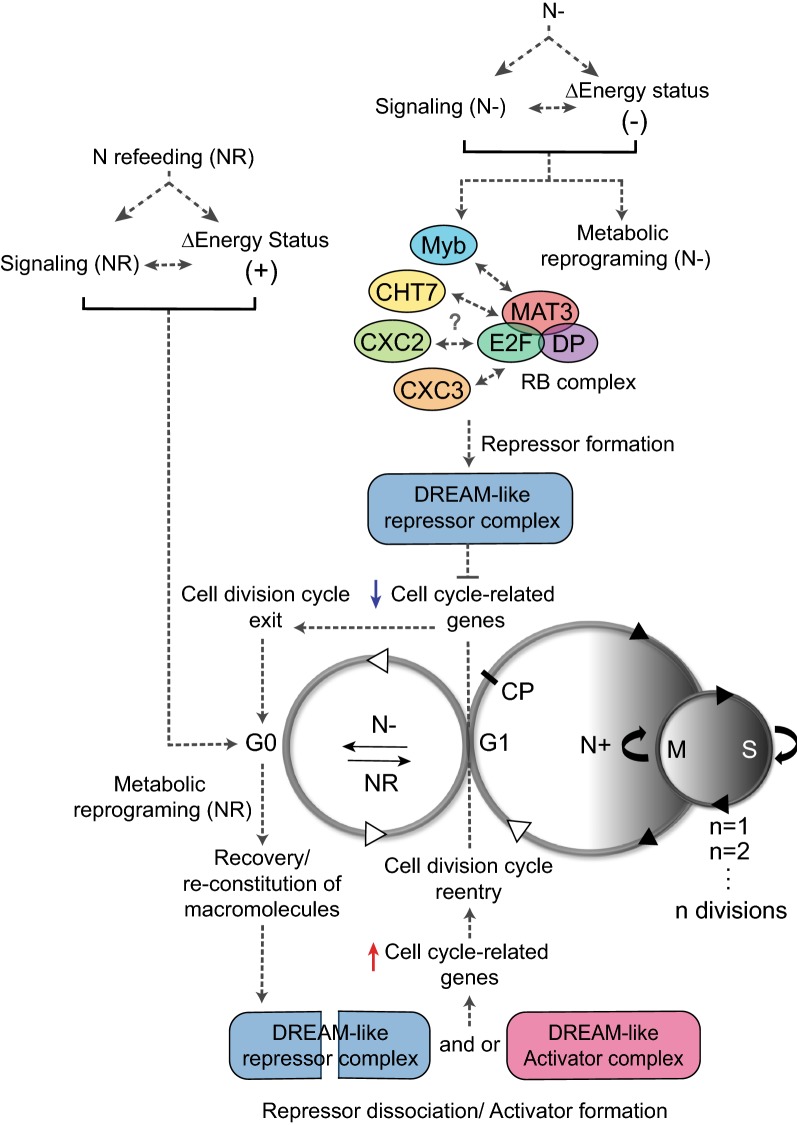
Proposed role of putative DREAM-like complexes in the nutrient-dependent life-cycle transitions of Chlamydomonas. Although the existence of DREAM-like (DP, RB, E2F and Myb-MuvB) complexes has not been confirmed for the algal lineage, the repression of genes related to the cell cycle and the cessation of growth and division with 1C (one copy) DNA content in the absence of N have been previously observed. Furthermore, some components of DREAM-like complexes are conserved in Chlamydomonas, including the RB pathway proteins (MAT3/RB, Cre06.g255450; E2F1, Cre01.g052300; DP1, Cre07.g323000), three CXC domain-containing proteins (CHT7, Cre11.g481800; CXC2, Cre08.g361400 and CXC3, Cre12.g550250; potential orthologs of mammalian LIN54, fly Mip120, worm lin-54, and Arabidopsis TCX5), and one Myb protein with three Myb-repeats (Myb3R, Cre12.g522400). The model of their hypothetical functions within the putative DREAM-like complexes in mediating the nutrient-dependent entry into and exit from quiescence (G0) is illustrated. The grey dotted lines are used to denote the hypothesized interactions. In line with the literature demonstrating their importance in the transcriptional regulation of cell cycle-dependent gene expression in other model organisms, the putative Chlamydomonas DREAM-like repressor complex is postulated to repress the genes associated with cell cycle progression during the post-mitotic or G1 phase prior to the passage of commitment point (CP) in response to N deprivation (N−), allowing the exit from active proliferation and entry into quiescence. Conversely, upon sensing the replenishment of N, the cells need to reinstate their capacity for energy capture and macromolecular synthesis. Once their metabolism is sufficiently restored to sustain further growth, the cell cycle-related genes are postulated to become activated by the dissociation of a DREAM-like repressor complex and or the formation of its activator counterpart, allowing the cells to fully exit from quiescence to reenter the cell division cycle. Although these complexes may also play a role in the progression of the cell division cycle itself, they are omitted from the model for the sake of simplicity. The plus and the minus signs next to the energy status represent energy sufficient and deficient states, respectively. Cell cycle-dependent steps are represented by black arrow heads, while the nutrient-dependent steps are represented by white arrow heads. N+: N-replete growth; N−: N deprivation; NR: N refeeding

Despite the recent advances in understanding the impact of nutrient availability on gene expression and metabolism of Chlamydomonas, the signaling pathways and molecular components enabling the entry into, maintenance of, and exit from quiescence remain largely unknown in photosynthetic eukaryotes. One potential regulatory component that may play a role in the nutrient-dependent life-cycle transitions of Chlamydomonas is the evolutionarily conserved multi-protein transcriptional regulatory complex known as DREAM (DP, RB, E2F and Myb-MuvB) (Fig. 3), although its presence in algae has yet to be confirmed. It is notably absent from yeast, but organisms from many evolutionary lineages including mammals [124–126], fruit flies [127, 128], worms [129, 130] and plants [131] utilize this structurally conserved module to coordinate the expression of the cell division cycle-dependent and development-specific genes in response to different cues present during quiescence, cell proliferation and differentiation, and organismal or sexual organ development [73, 74]. These complexes, whose activities are determined by the combinatorial presence of distinct components, are important for the context-dependent transcriptional regulation of cell cycle genes, whose protein abundance is largely determined at the transcriptional level [74]. The core components of DREAM complexes are conserved among species, which include retinoblastoma (RB) tumor suppressor proteins, adenovirus early gene 2 binding factor (E2F) family of transcription factors and their dimerization partners (DP), and the members of the multi-vulva class B (MuvB) complexes, which were initially identified through mutations that cause synthetic multi-vulva phenotypes in *Caenorhabditis elegans* [73, 74].

One of the best characterized core constituents of the metazoan MuvB complexes are the proteins that contain two tandem cysteine-rich motifs or domains that are collectively referred hereafter as the CXC domain. These CXC domain proteins include the mammalian LIN54 [124, 126], Drosophila Mip120 [127, 132], and *C. elegans* Lin-54 [129], and their CXC domains are necessary for the sequence-specific binding of DNA [132–135]. For instance, the CXC domain of mammalian LIN54 is known to directly bind the cis-regulatory element known as the cell cycle genes homology region (CHR) [133, 135], which is a primary promoter element involved in the regulation of G2/M phase genes [136–139]. Consequently, LIN54 is essential for the recruitment of both activator and repressor DREAM complexes to these sites [74]. The CHR consensus sequences, defined by TTYRAA, where Y and R represent pyrimidine and purine bases, respectively [135], are enriched in the promoter regions of MuvB-target genes, not only in humans [137–139] but also in flies [140] and worms [134]. Although a CXC domain protein, TCX5, is present in both the activator and repressor forms of Arabidopsis DREAM-like complexes, which play a crucial role in the regulation of G2/M phase-specific gene expression, its functional contributions within these complexes remain unknown, and the CHR elements are yet to be identified in plants [74, 131, 141]. Despite the ubiquitous presence of CXC domain-containing proteins in plants, the soybean CPP1 and maize CBBP remain some of the few cases where their CXC domains have been implicated in the binding of DNA [142, 143]. Regardless, in addition to TCX5, increasing numbers of studies are revealing the functions of CXC domain proteins in the regulation of the cell cycle and cell division of Arabidopsis. One such protein, TSO1, is involved in the control of the cell division cycle in meristems, shoots, and roots during plant development [144–149], and its closest paralogs, *SOL1* and *2* have recently been implicated in the regulation of stomatal cell division and fate transition [150].

Other transcription factors are also known to associate with the core complexes, but their presence is less conserved among different organisms. While recruitment of the forkhead box M1 (FOXM1) transcription factor to the MuvB complex is necessary for the full activation of G2/M phase genes in mammals, no forkhead transcription factors have been found in the orthologous complexes of flies, worms, and plants [74]. Furthermore, Myb-type transcription factors, which function as activators of gene expression both in mammals and flies, are apparently missing from the DRM complex of *C. elegans*, and the *C. elegans* DRM is thought to act primarily as a transcriptional repressor [74, 129]. This is in contrast to DREAM-like complexes of Arabidopsis, where a small family of Myb3R transcription factors with three Myb-repeats with activator or repressor function(s) regulate the expression of many G2/M phase-specific genes by interacting with the promoter sequence known as the mitosis-specific activator (MSA) element [131, 151–154]. Therefore, Myb3Rs play an important role in determining the direction of transcriptional regulation mediated by Arabidopsis DREAM-like complexes along with the corresponding E2Fs, and despite the seeming absence of the CHR elements in plants, the target promoter regions of Myb3R-containing DREAM-like complexes of Arabidopsis are found to be enriched in MSA and or E2F elements [131].

The Chlamydomonas genome also encodes some of the conserved components of DREAM complexes (Fig. 3), and their protein products have documented roles in the control of cell-size homeostasis, the cell division cycle, and quiescence. Chlamydomonas utilizes a homolog of the mammalian RB protein, MAT3, in coordination with the E2F1 transcriptional activator and its dimerization partner, DP1, to regulate cell size and cell cycle progression [61, 155, 156]. However, unlike its mammalian counterpart, the Chlamydomonas MAT3/RB complex is stably associated with chromatin, and the progression through the cell division cycle is thought to involve differential phosphorylation of the RB protein or the participation of additional activator or repressor proteins [156]. The *mat3*–*4* mutant is characterized by a misregulation of cell size homeostasis. Its cells are smaller in size than wild-type cells, because they pass the commitment point at a smaller volume and also undergo more rounds of the S/M cycle than the wild type [61, 155]. A novel class of cyclin dependent kinase, CDKG1, is one of the regulators responsible for coupling the mother cell size to the number of subsequent divisions, by phosphorylating MAT3 in a cyclin D-dependent manner [157]. A single Myb3R gene is encoded within the genome of Chlamydomonas, whose expression is upregulated when the light–dark synchronized Chlamydomonas cells go through division [158]. The coexpression network generated for Chlamydomonas using genome-wide transcriptomics conducted under a number of conditions, including nutrient deprivation, has also found this gene to coexpress or cluster closely with other cell cycle genes [159]. Despite the intriguing observation that the five repeats of the Arabidopsis MSA elements are found within the 600 bp upstream of the translational start site of Myb3R gene itself, no obvious enrichment of this motif has been observed in the promoter regions of cell cycle genes when the entire genome was used as a Ref. [158]. Furthermore, since none of the previously identified candidate cis-regulatory elements with a potential to regulate the diurnal transcription programs in Chlamydomonas appear to resemble the MSA motif [158, 160], further studies are needed to implicate Myb3R in cell cycle regulation or cell division.

In addition to the aforementioned genes, the Chlamydomonas genome encodes at least three proteins with annotated CXC domains. Although the literature on these proteins is scarce, one CXC domain protein in Chlamydomonas with characterized functions in the transcriptional regulation of quiescence-associated programs is the Compromised Hydrolysis of TAG 7 (CHT7) protein. The mutant ablated in CHT7 is impaired in its ability to remobilize TAG and shows delayed growth upon N or P resupply and rapamycin removal (following a period of N or P starvation or rapamycin treatment, respectively [71]). Decades ago, a similar delay in the resumption of growth was also observed for the *mat3* mutant during N refeeding [161]. Similarly to RB and related proteins, CHT7 proteins are located in the nucleus, although some are also observed in the cytosol [71]. No obvious defects in growth are detected in the *cht7* mutant during N-replete growth despite the large number of misregulated genes in this mutant under this condition. Of these genes, nearly 50%, including those involved in photosynthesis, flagellum assembly and autophagy, are expressed in *cht7* cells under N-replete conditions in a similar manner as in wild-type cells, which are subjected to N deprivation [71]. In addition, many genes involved in chloroplast-related functions, including photosynthesis, tetrapyrrole synthesis and plastid ribosomal protein synthesis, fail to reverse their expression upon N refeeding in *cht7* as would be typical for genes in the wild type [72]. Thus, CHT7 has been hypothesized as an apparent repressor, during N-replete growth and N refeeding of a subset of transcriptomic programs associated with N deprivation-induced quiescence. Furthermore, it was hypothesized that the complete exit from quiescence during N refeeding requires the repression of these programs by CHT7 [71, 72]. However, the mechanisms governing CHT7 activity remain unclear. Although the majority of CHT7 proteins of Chlamydomonas exist as part of a protein complex, the levels of CHT7 (per total protein) do not fluctuate with changes in N abundance, and the apparent size and abundance of the observed complex stay constant regardless of N availability [71]. Thus, further studies are needed to explore the molecular mechanisms by which the CHT7 complex affects quiescence, whether the CHT7 protein plays a direct or indirect role in the transcriptional regulation of cell cycle genes, or whether or not the Chlamydomonas CHT7 complex is functionally analogous to DREAM complexes in other organisms.

## Signaling networks linking the metabolic status to growth in Chlamydomonas: TOR and SnRK/CKIN pathways

**Fig. 4:**
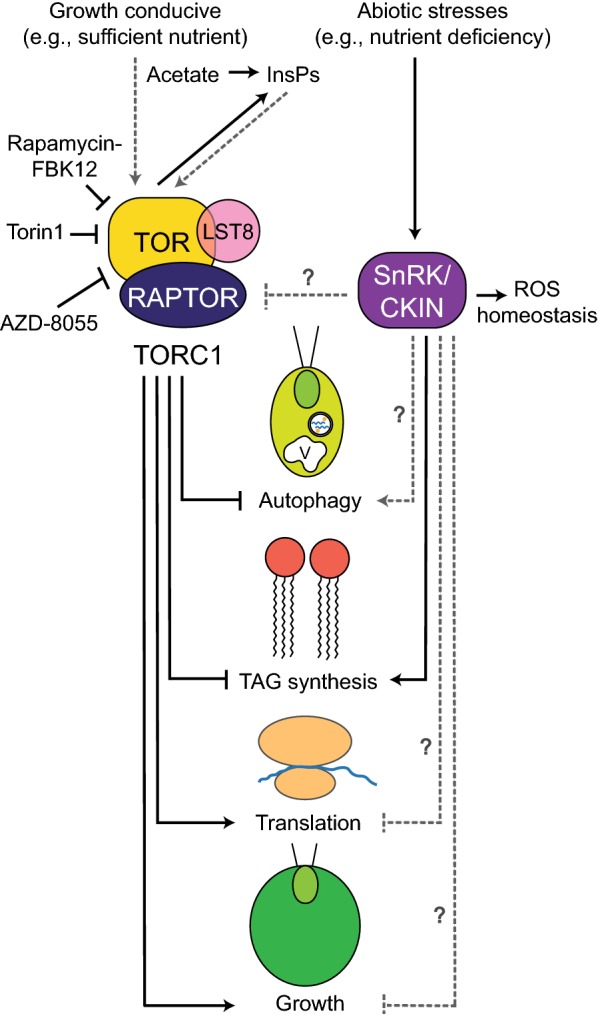
Working model of TOR and SnRK/CKIN family of kinases and their major functions in Chlamydomonas stress biology. Chlamydomonas rapamycin-sensitive TOR complex 1 (TORC1) consists of TOR, LST8 and RAPTOR. The inhibition of TORC1 by pharmacological means (rapamycin, AZD-8055 or Torin1) has enabled the studies of TOR pathway functions in Chlamydomonas, whereas the components of TORC2 have not been identified in photosynthetic organisms. When the conditions are conducive to growth (i.e., in the absence of abiotic stresses), the TORC1 complex promotes anabolic processes such as protein synthesis and therefore growth and represses stress-induced responses such as TAG synthesis and the induction of autophagy. TORC1 has also been shown to positively regulate the levels of inositol polyphosphates (InsPs), InsP_7_ and InsP_8_, to promote growth and inhibit TAG accumulation. Although the members of the SnRK and CKIN family of kinases in Chlamydomonas and microalgae are not well characterized to date, several studies have shown the functions of some members in the acclimation of cells to abiotic stresses, including the adjustment of reactive oxygen species (ROS), accumulation of TAG and sulfur (S) assimilation during sulfur deprivation. The relationships that are supported by experiments are shown in solid black lines, whereas the dotted grey lines with question marks represent the hypothesized regulatory links based on previous findings in other model organisms that require further studies in Chlamydomonas. The positive and negative regulatory relationships are represented by arrows and T-bars, respectively

Evolutionarily conserved signaling pathways playing a central role in the coordination of nutrient status with metabolism and cellular growth in eukaryotes, including photosynthetic organisms, are those involving the target of rapamycin (TOR) kinases and their antagonists, AMPK/Snf1/SnRK/CKIN kinases (Fig. 4). As suggested by their names, TOR1 and TOR2 kinases were first identified by a genetic mutant screen in yeast as targets of rapamycin [162], an antifungal and immunosuppressant compound isolated from the soil bacterium *Streptomyces hygroscopicus* [163, 164]. Whereas the treatment of wild-type yeast cells leads to the arrest of the cell cycle in the G1 phase, those with a mutation in the *TOR1* or *2* are resistant to the drug [162]. Although most organisms contain only a single TOR [165], the functional equivalents of two distinct multi-protein complexes discovered in yeast, TOR complex 1 (TORC1) and 2 (TORC2) [166–168], are present in many organisms [169–171]. Despite the identification of TOR and the conservation of TORC1 components, such as RAPTOR (regulatory-associated protein of TOR) and LST8 (lethal with SEC13 protein 8) in plants and algae [172–177], no obvious homologs of TORC2 components have been identified in organisms of the green-lineage, although its functional equivalent is postulated to exist [177–181]. Nevertheless, the primary functions of the TOR pathways and the mechanisms of TOR inhibition by rapamycin appear conserved in nearly all groups of organisms. When nutrients are ample, TOR complexes act as positive regulators of cellular growth by promoting anabolic processes such as nucleotide synthesis, transcription, ribosome biogenesis and translation, while inhibiting the opposing catabolic processes including mRNA degradation and autophagy [170, 179–183] (Fig. 4).

Rapamycin acts to inhibit TOR by interacting with a 12-kDa proline isomerase immunophilin known as FK506-binding protein (FKBP12) [162]. The FKBP12–rapamycin complex subsequently binds to the FRB (FKBP12–rapamycin binding) domain of TOR, leading to its inactivation by limiting the accessibility of its kinase domain to the substrate [184]. In opisthokonts, TORC1 is sensitive to rapamycin whereas TORC2 is not [166, 167, 185–187], largely owing to the presence of RICTOR (rapamycin-insensitive companion of TOR) in TORC2 which renders the FRB domain inaccessible to the FKBP12–rapamycin [188, 189]. In addition, since the FKBP12 proteins of many plant species are unable to stably associate with rapamycin, land plants are resistant or highly tolerant to the growth-inhibitory effects of rapamycin [172, 190–193]. In contrast, growth and cell cycle progression of Chlamydomonas are sensitive to rapamycin treatment, although to a lesser extent when compared to yeast or mammals due to the lower affinity of its FKBP12 protein to rapamycin [173]. As observed for other organisms, the Chlamydomonas TOR protein exists as part of a large molecular weight complex, and its single copy LST8 co-purifies with TOR and FKB12 in the presence of rapamycin, confirming the existence of Chlamydomonas TORC1 [175]. The Chlamydomonas LST8 plays a functionally analogous role to those of yeast and mammals, where the associations of the LST8 proteins with the kinase domains of respective TORs are necessary for their full catalytic activities [194, 195], and the seven WD-40 domains present within Chlamydomonas LST8 may have an additional function in facilitating the association of TORC1 with its various protein substrates [175]. Furthermore, some fractions of both TOR and LST8 appear to be peripherally associated with membranes of the endoplasmic reticulum (ER) system, particularly near the peri-basal body regions at the base of flagella, where the demand for protein synthesis is likely high [175, 196].

In animals and yeast, the network governed by TORC1 constitutes one of the major signaling pathways linking nutrient availability to the autophagic machinery, by the phosphorylation-mediated regulation of ATG (autophagy-related) proteins that orchestrate autophagy [197–199]. As discussed earlier, the activation of autophagy is a necessary cellular response to promote survival during starvation and the consequent establishment of a reversible state of quiescence. The ATG proteins are also conserved in Chlamydomonas [183], and the FKBP12–rapamycin mediated inhibition of TORC1 leads to an increased bleaching and vacuolization [173]. One such conserved ATG protein, ATG8 has also been demonstrated as an autophagy-specific marker in Chlamydomonas [93, 200]. In many organisms, the covalent attachment of phosphatidylethanolamine (PE) to ATG8 (known as lipidation) allows for the association of ATG8 proteins with the autophagosome vesicle until the fusion of the ATG8–autophagosome with the vacuole takes place [201]. Because the amount of ATG8 proteins is directly related to the number and size of autophagosomes, the levels of lipidated ATG8 and their altered cellular localization can be used as markers of active autophagy [202], which holds true also for Chlamydomonas [93, 200]. The treatment of Chlamydomonas cells with rapamycin leads to the accumulation of ATG8 and its lipidated forms, followed by their relocation to large punctate structures in the cytoplasm, indicating the inhibition of TORC1 as an important step in the activation of autophagy [93]. Moreover, the same ATG8-marker responses are induced upon subjecting the cells to nutrient starvation and oxidative or ER stresses, illuminating the role of the TORC1 pathway in regulating stress-induced autophagy of this alga [93, 203].

In opisthokonts as well as plants, the mechanisms by which TORC1 promotes protein synthesis are known [204–208]. For instance, in mammals, the direct phosphorylation and activation of S6 kinase (S6K) by TORC1 leads to the S6K-dependent phosphorylation of the ribosomal protein S6, resulting in increased rates of translation initiation [204, 207]. The TOR–S6K pathway is also conserved in plants, and the translation initiation of cytosolic S6 ribosomal protein in Arabidopsis is likewise regulated by this pathway [192, 208–210]. The Arabidopsis TOR kinase also promotes plastid ribosomal biogenesis by upregulating the transcription and translation of genes and mRNAs, respectively, for nuclear encoded plastid ribosomal proteins [208]. Although Chlamydomonas TOR kinase is implicated in the regulation of de novo amino acid synthesis [211, 212], and rapamycin treatment is also known to inhibit protein synthesis in this alga [213], the signaling pathways downstream of TOR controlling protein synthesis are generally less well characterized in algae. However, TOR-dependent phosphorylation sites were also recently identified in S6K and ribosomal S6 protein of Chlamydomonas through phosphoproteomic studies of cells treated with rapamycin, AZD-8055, or Torin1 [214–217], and TORC1-mediated phosphorylation of the ribosomal S6 protein at serine-245 was shown to be regulated by N as well as P availability in Chlamydomonas [218, 219]. Furthermore, a recent study has begun to establish the regulatory link between P availability and TORC1-signaling Chlamydomonas [219]. Using the phosphorylation of ribosomal S6 protein as a marker of TORC1 activity, it was shown that TORC1 becomes inhibited following P starvation likely through a drastic reduction in the abundance of LST8 proteins, which are necessary for the activity of TOR complexes [166, 194, 195, 219]. Therefore, it is increasingly evident that the inhibition of TORC1 and the deprivation of nutrients both trigger similar cellular processes and stress responses, not only in yeast where the role of TOR pathways in the coordination of nutrient status to cellular growth is firmly established [220–223], but also in algae [35, 224–227]. In addition to the cessation of growth, the activation of autophagy and the reduction in protein synthesis that occur upon TORC1 inhibition and nutrient starvation, the repression of TORC1 and N shortage both induce the formation of TAG-rich lipid droplets in various species of algae [35, 224–226]. In Chlamydomonas and the red alga *Cyanidioschyzon merolae*, the repression of TORC1 pathways by pharmacological means (rapamycin, AZD8055, or Torin1) has been shown to result in the upregulation of key enzymes involved in TAG biosynthesis, such as glycerol-3-phosphate acyltransferase (GPAT) and DGAT [224, 225]. Consistent with these observations, the accumulation of TAG and starch is also reported for Arabidopsis seedlings with inducible repression of TOR [228].

Although the molecular mechanisms by which TOR pathways regulate lipid metabolism or TAG accumulation are currently not well known in algae, a recent genetic screen for Chlamydomonas mutants with increased sensitivity to rapamycin has identified the *VIP1* locus, suggesting a relationship between inositol polyphosphates (InsPs), TAG accumulation, and TOR [229]. The *VIP1* gene encodes a kinase responsible for the pyrophosphorylation of InsP_6_ to yield InsP_7_ and InsP_8_, which are important signaling molecules [229]. The *vip1*-*1* mutant has decreased levels of InsP_7_ and InsP_8_, slower growth and increased levels of TAG during mixotrophic growth in the presence of acetate [229]. A similar reduction in the InsP_7_ and InsP_8_ content was observed for rapamycin-treated wild-type cells, further suggesting a link between InsPs, TAG, and the TOR pathway [229]. In addition, the expression profiles of thousands of genes are reported to change in response to the rapamycin treatment of Chlamydomonas cells [230], and they appear to at least partially resemble the transcriptional program associated with nutrient starvation. Following rapamycin treatment, where genes involved in autophagy, vacuolar function, amino acid metabolism and transport tend to be upregulated, genes involved in processes that require a robust anabolic metabolism, e.g., nucleotide synthesis to sustain DNA replication and the cell cycle become downregulated [230]. The decrease in the transcript levels of cell cycle-related genes in response to rapamycin is not only consistent with the observed inhibition of growth following TORC1 inactivation in Chlamydomonas [173, 211], but also with studies in Arabidopsis, where the expression of E2Fa and E2Fb targets with central roles in the regulation of cell cycle is activated by the TORC1-mediated phosphorylation and repressed upon TORC1 inhibition [231, 232]. Arabidopsis TORC1 was also recently shown to phosphorylate and inhibit a member of the dual-specificity tyrosine phosphorylation-regulated kinase (DRYK) family, AtYAK1 an orthologue of Yet Another Kinase 1 in yeast), which acts as a negative regulator of plant growth [233–235]. Under conditions where TORC1 is inactive, the repression on AtYAK1 is lifted, and AtYAK1 activates plant-specific CDK inhibitors, SMR (Siamese-related) proteins, to negatively regulate cell cycle progression [235]. Yeast YAK1 and its metazoan orthologs, mammalian DYRK1A and fly Minibrain kinases, also have known roles in inhibiting proliferation [236–242]. The mammalian DYRK1A upregulates the expression of the gene encoding CDK inhibitor, *CDKN1B* (also known as *p27*_*KIP1*_) [239]. The DYRK1A-mediated phosphorylation of CDKN1B, in addition to cyclin D1 and D3, promotes CDKN1B stabilization, cyclin D degradation and consequently cell cycle exit [237, 240]. Furthermore, the mammalian DYRK1A also phosphorylates LIN52, a component of the MuvB core, to facilitate DREAM complex formation and to promote entry into quiescence or senescence [241]. In these contexts, it may be worthy to note that Chlamydomonas also has an ortholog of YAK1 named TAR1 (TAG accumulation regulator 1), another green-lineage-specific DRYK kinase, DYRKP, and other DRYK-related kinases [243, 244]. While both TAR1 and DYRKP have been shown to regulate the accumulation of TAG and for DYRKP, of starch during N and S deprivation [243, 244], their potential role in the nutrient-dependent regulation of the Chlamydomonas cell cycle is not yet clear.

In animals and yeast, AMP-activated kinase (AMPK) and sucrose nonfermenting 1 (Snf1) kinase, respectively, are central signaling components that are activated by nutrient limitation and other stresses, and act antagonistically to TORC1 [245–249]. The orthologs of AMPK/Snf1 are also conserved in plants and algae, and they are known as Snf-related kinases (SnRKs) in Arabidopsis and sometimes referred to as CKINs (Chlamydomonas kinases) in Chlamydomonas [179, 250]. In general, the activated AMPK/Snf1/SnRK/CKIN signaling pathway promotes cellular survival and cessation of growth during stress by upregulating catabolic processes to generate more energy and downregulating growth-promoting processes to consume less energy [179, 247, 249–253] (Fig. 4). In mammalian cells, AMPK is known to inactivate TORC1 in response to energy and nutrient stresses by phosphorylating one of its constituents, RAPTOR, and by the subsequent recruitment of 14-3-3 proteins [254]. In addition to its inhibitory effect on TORC1, AMPK facilitates the arrest of the cell cycle during the G1 phase prior to the replication of DNA by upregulating and stabilizing the levels of CDK inhibitors, CDKN1A (also known as p21_WAF1_) and CDKN1B (also known as p27_KIP1_), respectively [255–257]. The AMPK also promotes the initiation of autophagy, upregulates the uptake of glucose and fatty acids, and facilitates the breakdown of these molecules by the activation of glycolysis and fatty acid oxidation, respectively [249, 252]. In a similarly opposing manner to TORC1, AMPK acts to inhibit the biosynthesis of nearly all macromolecules, including proteins, ribosomal RNA, lipids, and carbohydrates by the direct phosphorylation of various key components and regulatory factors of these anabolic pathways [249].

The SnRK family of kinases in Arabidopsis is classified into three subfamilies. The SnRK1 subfamily represents the smallest group with three genes (*SnRK1α1*–*3*), and they have the greatest similarity to the yeast Snf1 [258]. The SnRK1 family of genes coordinates the energy and redox homeostasis of plants in response to a plethora of growth-inhibiting stresses and regulates a broad range of metabolic pathways through the phosphorylation of the key enzymes or transcription factors to improve stress tolerance and promote survival [259–261]. In this context, the members of the SnRK1 family act to inhibit highly anabolic processes such as protein synthesis and proliferation, while activating stress-induced responses such as gluconeogenesis and starch synthesis in plants [179]. The antagonistic activities of SnRK1α1 towards TORC1 have also been demonstrated in Arabidopsis by its ability to interact with and phosphorylate RAPTOR1B [262]. Although a complete knockout of *SnRK1* genes results in embryonic lethality in Arabidopsis [262], similarly to the knockout mutants of *TOR* [172], inducible *amiRNA::SnRK1α2* transgenic plants in the *snrk1α1* mutant background show a hyper-phosphorylation of ribosomal protein S6, indicating their crucial role in the suppression of TORC1 and the downregulation of translation [262]. The SnRK2 and 3 subfamilies in Arabidopsis are also reported to function in the adaptation of plants to a wide range of abiotic stresses, including drought, flood, cold, salinity, and nutrient scarcity [263]. Although the characterization of the SnRK family in algae lags behind, a number of studies in Chlamydomonas have suggested the role of SnRKs/CKINs in the cellular response to abiotic stresses, including cold [264] and shortages of S [89, 265, 266] and N [47]. In Chlamydomonas SAC1 and SnRK2.2 have been implicated in the regulation of TAG synthesis during S deprivation by modulating the expression of *DGTT1–4* [267]. Indeed, the biotechnical implications of this signaling pathway have recently prompted the genome-wide identification of 22 CKIN proteins in Chlamydomonas as orthologs of plant SnRKs [250]. Whether the orthologous DREAM complex components in Chlamydomonas are targeted by members of a TOR or SnRK/CKIN signaling cascade to relay cellular nutrient status and to regulate the transitions between cell division and quiescence cycles, remain to be elucidated. However, these studies are starting to shed light on the significance of signaling pathways involving TOR and SnRK/CKIN in the coordination of nutrient availability, energy metabolism, and cellular growth in photosynthetic organisms. This emerging knowledge provides an essential basis for the further exploration of these signaling networks and assessment of their bioengineering potential in microalgae.

## Concluding remarks

Given the ongoing biotechnological interests in algae, increasing numbers of studies are giving rise to a systems-level understanding of how various algal species respond to nutrient starvation, and how the metabolic pathways leading to the accumulation of TAG are regulated. Although the knowledge of transcriptomic and metabolic changes accompanying nutrient shortage and the entry into quiescence in algae continues to improve and evolve, the signaling and molecular components coordinating metabolism, energy status and cell division cycle are still not well-understood. The trade-offs between growth and the accumulation of economically valuable compounds thus continue to hinder the directed metabolic engineering of algae for biofuels and the commercial viability of utilizing algae as a chassis for the synthesis of high-value products. However, a better understanding of the controls of the cell division cycle in response to nutrient shortage and the signaling pathways coupling the cellular growth to energy and lipid homeostasis has the potential to improve the future metabolic engineering strategies of algae. Indeed, emerging evidence suggests that the manipulation of signaling pathways, such as TOR, represents a viable approach to increasing the lipid productivity in algae with little to no growth penalties [35, 268]. Thus, further studies of the signaling networks and the downstream components mediating and linking these biological processes are crucial in bridging a critical knowledge gap, which currently prevents us from achieving the optimal balance between the production of biofuels and biomass in algae employing simple and robust culturing conditions.

## Data Availability

Not applicable.

## Abbreviations

ACC1: acetyl-CoA carboxylase 1
AMPK: AMP-activated kinase
ATG: autophagy-related protein
BN-PAGE: blue native polyacrylamide gel electrophoresis
C: carbon
CDK: cyclin-dependent kinase
CDKN1A/B: cyclin-dependent kinase inhibitor 1A/B
CHR: cell cycle genes homology region
CHT7: Compromised Hydrolysis of Triacylglycerols 7
CKIN: Chlamydomonas kinase
CP: commitment point
DAG: diacylglycerols
DGAT/DGTT: diacylglycerol acyltransferase type 1/type 2
DP: dimerization partner
DREAM: DP, RB, E2F, Myb-MuvB
DYRK: dual-specificity tyrosine phosphorylation-regulated kinase
E2F: adenovirus early gene 2 binding factor
ER: endoplasmic reticulum
FAS1/2: fatty acid synthase 1/2
FKBP12: FK506-binding protein
FOXM1: forkhead box M1
FRB: FKBP12–rapamycin binding
GPAT: glycerol-3-phosphate acyltransferase
InsP: inositol polyphosphate
LIP1: lipase 1
LST8: lethal with SEC13 protein 8
MGDG: monogalactosyldiacylglycerols
MSA: mitosis-specific activator element
MuvB: multi-vulva class B
N: nitrogen
P: phosphorus
PDAT: phospholipid:diacylglycerol acyltransferase
PE: phosphatidylethanolamine
PGD1: Plastid Galactoglycerolipid Degradation 1
RAPTOR: regulatory-associated protein of TOR
RB: retinoblastoma tumor suppressor protein
RICTOR: rapamycin-insensitive companion of TOR
ROS: reactive oxygen species
S: sulfur
S6K: S6 kinase
SMR: Siamese-related
Snf1: sucrose nonfermenting 1
SnRK: sucrose nonfermenting-related kinase
TAG: triacylglycerols
TAR1: TAG accumulation regulator 1
TOR: target of rapamycin
TORC1/2: target of rapamycin complex 1/2
YAK1: Yet Another Kinase 1
